# A novel deep learning technique for multi classify Alzheimer disease: hyperparameter optimization technique

**DOI:** 10.3389/frai.2025.1558725

**Published:** 2025-04-24

**Authors:** A. S. Elmotelb, Fayroz F. Sherif, A. S. Abohamama, Mahmoud Fakhr, Amr M. Abdelatif

**Affiliations:** ^1^Department of Computer Science, Faculty of Computers and Informatics, Zagazig University, Zagazig, Egypt; ^2^Computers and Systems Department, Electronics Research Institute (ERI), Cairo, Egypt; ^3^Department of Computer Science, Faculty of Computers and Information, Mansoura University, Mansoura, Egypt; ^4^Department of Computer Science, Arab East Colleges, Riyadh, Saudi Arabia

**Keywords:** Alzheimer’s disease phases, multi-classification, deep learning, hyperparameters, ResNet152V25

## Abstract

A progressive brain disease that affects memory and cognitive function is Alzheimer’s disease (AD). To put therapies in place that potentially slow the progression of AD, early diagnosis and detection are essential. Early detection of these phases enables early activities, which are essential for controlling the disease. To address issues with limited data and computing resources, this work presents a novel deep-learning method based on using a newly proposed hyperparameter optimization method to identify the hyperparameters of ResNet152V2 model for classifying the phases of AD more accurately. The proposed model is compared to state-of-the-art models divided into two categories: transfer learning models and classical models to showcase its effectiveness and efficiency. This comparison is based on four performance metrics: recall, precision, F1 score, and accuracy. According to the experimental results, the proposed method is more efficient and effective in classifying various AD phases.

## 1 Introduction

A progressive brain disease that affects memory and cognitive function is Alzheimer’s disease (AD). It is the main reason for dementia that affects aged people, impacting millions throughout the world. The disease’s spread is predicted to explode in the next few years, making it a huge public health issue. Early detection of AD is critical for effective treatment and disease control. Recently, there has been a high interest in applying artificial intelligence (AI) models to predict and detect the early symptoms of AD, obtaining promising results in accuracy and early treatment. The use of artificial intelligence techniques in predicting Alzheimer’s disease is a recent interest in the field of computer science.

AD progresses in phases (mild, moderate, and severe), each with its symptoms and nature (Alzheimer’s Association, n.d.; Li et al., 2024; By Mayo Clinic Staff, 2023). In the early phase, people may suffer from mild cognitive impairment (MCI), which shows slight changes in their capacity to reason and remember. The second phase of dementia lasts the longest and comes with noticeable symptoms like confusion, behavior changes, and difficulty speaking. While in the late phase, people lose most of their mental and physical abilities, may not be able to talk, and need help with everyday tasks all the time. The treatments vary from person to person, and each phase’s length is not fixed (Jraba et al., 2024; Winchester et al., 2023).

Detecting AD involves several methods, such as automated systems, brain imaging, and machine learning techniques. Neuroimaging and machine learning are the main tools used to diagnose AD. Important brain imaging techniques include Magnetic Resonance Imaging (MRI), PET (positron emission tomography), Functional MRI (fMRI), and Diffusion Tensor Imaging (DTI) (Afzal et al., 2021; Shukla et al., 2023).

AI tools like Support Vector Machines, Bayesian Classifiers, and Deep Learning are used with these brain imaging techniques to make diagnoses more accurate. These tools inspect brain data to find patterns associated with AD, assisting in early detection and tracing of disease progress. The combination of brain imaging and AI shows promise (Alsubaie et al., 2024; Logan et al., 2021). Despite the limitations and drawbacks associated with these tools, which can be summarized in the following:

- The complexity of neuroimaging data.
- Lacking automated approaches (Aberathne et al., 2023).
- Lacking effective diagnostic methods that help in the early diagnosis of AD.
- Data acquisition and collection (Arafa et al., 2022).
- Biomarker limitations (Dubois et al., 2021).

The main goal of this research is to find a suitable and effective framework for classifying AD phases and overcoming data limitations and computational resources. We propose a mathematical model called the HPO (Hyperparameters Optimization) model, which will be discussed later, applying its outputs to a pre-trained model such as the ResNetV2 model, especially (ResNet152V2), to build a framework for classifying AD phases with the focus on overcoming the challenges of data limitations and computational resources.

The main contribution of this paper can be summarized as follows:

- Proposing a new HPO model for finding hyperparameters of deep learning techniques to improve their accuracy in classifying AD phases.
- A multi-classification of AD phases: MildDemented, ModerateDemented, NonDemented, and VeryMildDemented with enhanced results.
- Proof of the strength of ResNetV2 models in medical images, especially in AD classification.

The paper is organized as follows: Firstly, it will discuss related work and its limitations and drawbacks. Secondly, it will highlight the importance of deep-learning models and the significance of hyperparameters. Then, it will demonstrate and test the proposed approach using AD datasets. Finally, it will present results that indicate the effectiveness of the proposed approach.

## 2 Literature review

A variety of research was carried out using AI in detecting and diagnosing AI to better understand its nature and treatment process. The research covered all related topics of the disease, including early detection, using AI for the prediction of AD, tracing AD progression, and the combination of AI with neuroimaging for diagnosing processes.

One research study used automated processes and machine learning techniques to identify AD phases with over 95% accuracy. Better feature extraction and classification techniques are required, as biomarker approaches perform poorly in multi-group classification even while they perform well in binary classification (Shojaei et al., 2023).

Moreover, in this paper, the collective AI for detecting and diagnosing AD was investigated, showing its importance. However, it showed great promise and results, but it faced complications and challenges concerning data diversity and integration. That caused complications in model training and evaluation (Neshat et al., 2024).

In the systematic review that focused on natural language processing for AD detection, it was concluded that it wasn’t as efficient and methodical as the demographic variables in patients were unbalanced and lacked performance standard metrics (Petti et al., 2020).

Another study focused on using lightweight deep-learning models for AD detection and diagnosis using MRI data. As the proposed model was uncomplicated and simple, it only had seven layers; it could be implemented and applied in real-time applications. Additionally, it underlined the shortcomings and complexity of conventional models, highlighting the importance of lightweight models that may offer reliable and efficient alternatives. The suggested approach performed well in the binary classification of AD, but the results were unsatisfactory in the multiclassification of AD phases (El-Latif et al., 2023).

In Li and Yang (2021), three models were compared and evaluated: Support Vector Machine (SVM) and two deep learning algorithms (3D-VGGNet and 3D-ResNet). Using Grad-CAM for visualization, it successfully detected disease regions and obtained excellent accuracy in binary classification (AD vs. normal). However, its application in a variety of clinical environments is limited by dependence on high-quality MRI data.

Recent studies indicated that hierarchical binary classifiers improved by Ant Colony Optimization (ACO) performed better in mechanical problems classification, showing potential applications in AD classification. But it could not be generalized across datasets and may be restricted due to the reliance on specific optimization techniques (Vinodha and Gopi, 2024).

In addition, a review of imbalanced data classification emphasized the challenges posed by uneven class distributions, proposing various strategies such as algorithmic adjustments and data rebalancing techniques. However, the major challenges and drawbacks of these approaches are extensive preprocessing, computation density, and resource requirements. Which leads to limitations of implementation within real-time applications and environments (Yang et al., 2024).

In addition, these models were proposed and implemented depending on high-quality data and needed extensive feature extraction engineering, which might be considered another drawback and challenge. Besides, many studies were proposed and addressed AD detection theoretically and were not practically adequate and interpretable. Which is critical for clinical applications (Park et al., 2023). While the use of AI in AD research shows great potential, several drawbacks need to be addressed. These include the need for high-quality and standardized datasets, ethical considerations in the use of AI, and the potential limitations of AI in predicting disease progression and finding a cure. Finally, the advancements in binary and multi-classification techniques for AD have shown promising results, but challenges such as data imbalance, model complexity, and interpretability persist, necessitating further research and innovation in this critical area of health informatics.

### 2.1 Deep learning models hyperparameters

Hyperparameters in deep learning models are important since they define the network architecture and how it is trained. These parameters are specified before the training process and include variables related to the training method, such as epochs, iterations per epoch, dropout rate, batch size, and optimizer (Jafar and Lee, 2023).

The selection of hyperparameters considerably influences the performance of the deep learning model. So, finding the right set of hyperparameters is essential for achieving an optimal deep learning model. Hyperparameter tuning, which involves searching the hyperparameter space for the best combination of values, is a critical step in the model development process. Various methods, such as manual search, grid search, random search, and Bayesian optimization, are used to find the optimal set of hyperparameters, ensuring the model’s effectiveness and efficiency (Fabrizio et al., 2021; Ghazal and Issa, 2022; IBM, n.d.; Subramanian et al., 2022).

The importance of hyperparameters in deep learning models is summarized below (Arnold et al., 2024; Naushad et al., 2021; Shojaei et al., 2023):

*Learning rate* indicates how rapidly a network adjusts its parameters. A high learning rate may result in unstable training, whereas a low learning rate may cause slow convergence.
*Batch size* determines how many samples are utilized in each training iteration. A small batch size may produce noisy gradients, whereas a large batch size may cause weak convergence.
*The activation function* determines the model’s nonlinearity. Different activation functions may be better suited to different sorts of data.
*Dropout* helps minimize overfitting by randomly removing units during training. The dropout rate is the probability of dropping out of each unit.
*Optimizer* determines the algorithm for updating the model’s parameters. Certain optimizers, like Adam and SGD, may be better suited to certain sorts of data.
*Early stopping* prevents overfitting by halting the training process when the validation loss no longer improves. A hyperparameter specifies how many epochs to wait before ending.

Finally, each hyperparameter in deep learning models has a significant impact on the model’s performance. Each hyperparameter’s importance is determined by the individual problem and data being used. Thus, hyperparameter tuning is required to determine the optimum set of hyperparameters for a specific problem.

### 2.2 ResNet152V2 model

ResNet152V2 is a deep learning model that belongs to the residual network family. Using residual connections, the ResNet152V2 model efficiently trains very deep networks with 152 layers (see Table 1) (Shaziya and Zaheer, 2021). Because of its great accuracy and efficiency, it has been used in many different applications, such as medical diagnosis and image classification. The ResNet152V2 model has 71,177,348 parameters, including 143,744 that are non-trainable and 71,033,604 that are trainable. Compared to developing a model from scratch, this pre-trained model helps to achieve acceptable accuracy more rapidly since it contains starting weights (Ibrahim et al., 2021; Nagarathna et al., 2023).

**Table 1:** The general structure of ResNet152v2 along with the function of these layers.

Layer	Description
Input Layer	Accepts image as input.
Convolutional Layers	Initial convolutional layers extract low-level features. Batch normalization layers normalize activations. ReLU activation function introduces non-linearity.
Residual Blocks	Building blocks with shortcut connections
Bottleneck Blocks	Reduce computation and memory requirements.
Global Average Pooling	Reduce spatial dimensions of feature maps.
Fully Connected Layers	Receive global average pooled features for classification. The number of layers depends on the specific task.
Output Layer	Products predicted class probabilities.

## 3 Material and method

In this paper, we propose a model called HPO that will be used to expect epoch number, batch size, and dropout factor based on input size and expected target accuracy, as we noticed most recent studies adjust these parameters based on the babysitting approach, i.e., trial and error, which leads to using a huge number of computations for training models and is time-consuming.

### 3.1 Hyperparameters optimization (HPO)

Traditionally, there were many methodologies for finding the optimal configuration of these parameters: babysitting approach, grid search, random search, and Bayesian optimization (Farag et al., 2021; Ibrahim et al., 2021; Jafar and Myungho, 2020), they had many drawbacks. The main ones were time and resource consumption, and the vast number of computations.

Therefore, we study the nature of the most used heuristic techniques: Bayesian optimization and random search (Ali et al., 2023; Bai et al., 2023; Turner et al., 2021) investigation of their limitations and drawbacks; as Bayesian optimization was complex structure and computations overhead, and random search however it was simple but might not find the optimum configurations of hyperparameters.

**Figure 1:**
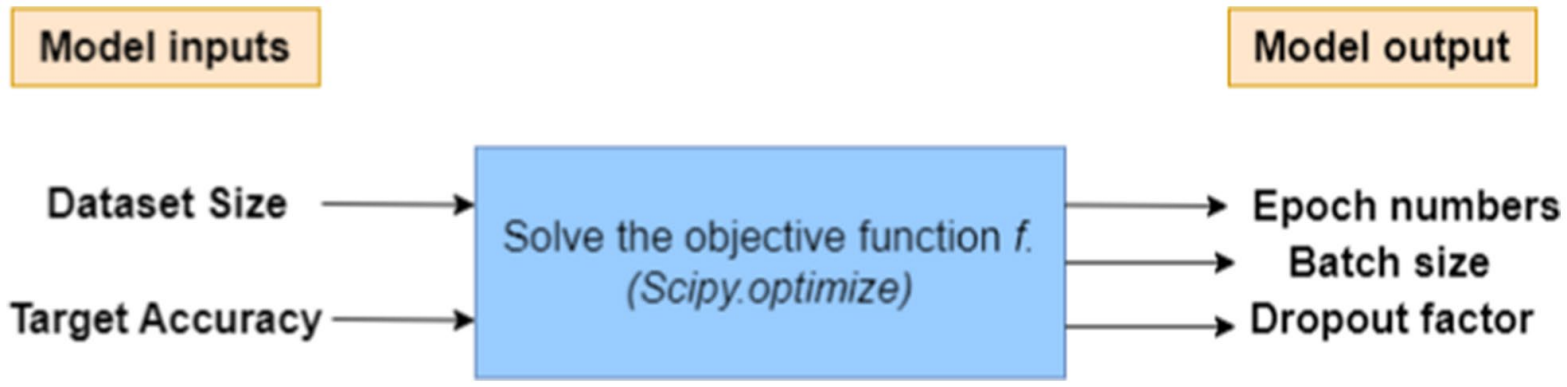
Proposed HPO model that estimates the hyperparameters.

Then create a mathematical model called the HPO model, see Figure 1, that estimates the number of epochs, batch size, and dropout factor depending on the input size and target accuracy. The proposed mathematical model (HPO) is supposed to offer preliminary estimations and may not correctly reflect all real-world issues, but we apply the approximation theory (Deo et al., 2020; Elbrächter et al., 2021; Kamath et al., 2021; Leluc, n.d.). The actual performance of a model may differ based on its difficulties and dataset.

The proposed HPO model works as the model inputs and processes these inputs to solve the objective function then computes the model output as follows:

*Model’s input:* Dataset size and Target accuracy.
*Model’s output:* a desirable set of hyperparameters (epoch number, batch size, and dropout factor).

Let us define the following variables:

*N* = Dataset size (number of samples in the dataset).
*Acc* = Target accuracy (e.g., 0.95 for 95% accuracy), $Acc\in R^{+\text{.}}$
*En* = Number of epochs, En∈N
*Bs* = Batch size, Bs∈N
*Df* = Dropout factor (a value between 0 and 1)

#### 3.1.1 Assumptions recap

- We assume a simple training model based on a feedforward neural network.
- To keep things simple, the relation between input size *N* and number of epochs *En* is linear En∝N .
- The batch size *Bs* is inversely related to the square root of the input size, this concept is supported by various discussions in machine learning literature $\propto \frac{1}{\surd N}$ .
- The dropout factor *Df* is calculated and influenced depending on the target accuracy, i.e., Higher target accuracy would likely correspond to lower dropout (stronger model) $Df\in \left[0,1\right]$ .
- We use the SLSQP (Sequential Least Squares Programming) implemented within the SciPy library (Brownlee, 2021; Rayhan and Kinzler, 2023) which is a mathematical library in Python to solve our objective function after adjusting the initial values of the hyperparameters.

The objective function f can be as follows:

(1) $$f=\frac{Acc}{En\times Bs\times \left(1-Df\right)}$$

#### 3.1.2 Validating the relationship

- Relation between *En, N* and *f*: we assume the number of epochs increases when dataset size increases, where *k* is a constant positive integer number, i.e., $k\in Z^{+}$ .

(2) En=kN

- Relation between *Bs, N* and *f*: we assume that batch size decreases with the square root of the dataset size, where *c* is a constant positive integer number, i.e., $c\in Z^{+}$

(3) $$Bs\propto \frac{c}{\sqrt{N}}$$

This shows that *f* is inversely proportional to √N . As the dataset size increases, the objective function *f* decreases because more epochs and smaller batch sizes are needed to process the larger dataset, thus increasing the computational cost.

- Relation between *Df, N* and *f*: As *Df* increases, the term (*1 − Df*) decreases, making the denominator larger and thus reducing *f*.

Substitution in from Equations 2, 3 in Equation 1

(4) $$f=\frac{Acc\times \surd N}{kN\times c\times \left(1-Df\right)}$$

*Theorem 1*: The objective function, *f*, can be obtained from the relation equation

$$f=\frac{Acc}{En\times Bs\times \left(1-Df\right)}$$

wheref,

- Has inverse relation with *En*, i.e., $f\propto \frac{1}{En}$
- Has inverse relation with *Bs*, i.e., $f\propto \frac{1}{Bs}$
- Has inverse relation with *Df*, i.e., $f\propto \frac{1}{\left(1-Df\right)}$

**Proof of Theorem 1:** We evaluate the objective function *f* sensitivity to hyperparameter changes by calculating partial derivatives. To find the partial derivatives of a function

$$\mathrm{Since}\;f\left(x\right)=\frac{g\left(x\right)}{h\left(x\right)},f^{\prime }\left(x\right)=\frac{g^{\prime }\left(x\right)h\left(x\right)-g\left(x\right)h^{\prime }\left(x\right)}{{\left(h\left(x\right)\right)}^{2}}$$

- Partial derivatives with respect to *En*: to find the partial derivatives of Equation 1 with respect to *En*.
$$\mathrm{let},g\left(En\right)=Acc,h\left(En\right)=En\times Bs\times \left(1-Df\right)$$

Step 1: Differentiate $g\left(En\right)$

Since *Acc* is treated as a constant with respect to *En*:

$$g^{\prime }\left(En\right)=0$$

Step 2: Differentiate $h\left(En\right)$

$$h\left(En\right)=En\times Bs\times \left(1-Df\right)$$

Here both *Bs* and *(1- Df)* are treated as constant with respect to *En* then

$$h^{\prime }\left(En\right)=Bs\times \left(1-Df\right)$$

$$\frac{\partial f}{\partial En}=\frac{0\times h\left(En\right)-g\left(En\right)\times h^{\prime }\left(En\right)}{{\left(h\left(En\right)\right)}^{2}}$$

$$\frac{\partial f}{\partial En}=\frac{-Acc\left(Bs\times \left(1-Df\right)\right)}{{\left(En\times Bs\times \left(1-Df\right)\right)}^{2}}$$

Simplify it:

$$\frac{\partial f}{\partial En}=-\frac{Acc}{En^{2}\times Bs\times \left(1-Df\right)}$$

⸪ derivative is negative

∴Enincreases whenfdecreases…. (Equation 1).

Confirming that the function penalizes larger numbers of epochs.

- Partial derivatives with respect to *Bs*: to find the partial derivatives of Equation 1 with respect to *Bs*.
$$\mathrm{let},g\left(Bs\right)=Acc,h\left(Bs\right)=En\times Bs\times \left(1-Df\right)$$

Step 1: Differentiate $g\left(Bs\right)$

Since *Acc* is treated as a constant with respect to *Bs*:

$$g^{\prime }\left(Bs\right)=0$$

Step 2: Differentiate $h\left(Bs\right)$

$$h\left(Bs\right)=En\times Bs\times \left(1-Df\right)$$

Here both *En* and (1−*Df*) are treated as constant with respect to *Bs* then

$$h^{\prime }\left(Bs\right)=En\times \left(1-Df\right)$$

$$\frac{\partial f}{\partial Bs}=\frac{0\times h\left(Bs\right)-g\left(Bs\right)\times h^{\prime }\left(Bs\right)}{{\left(h\left(Bs\right)\right)}^{2}}$$

$$\frac{\partial f}{\partial Bs}=\frac{-Acc\left(En\times \left(1-Df\right)\right)}{{\left(En\times Bs\times \left(1-Df\right)\right)}^{2}}$$

Simplify it:

$$\frac{\partial f}{\partial Bs}=-\frac{Acc}{Bs^{2}\times En\times \left(1-Df\right)}$$

⸪ derivative is negative

∴Bsincreases whenfdecreases… (Equation 2).

The objective function appropriately penalizes large batch sizes.

- Partial derivatives with respect to *Df:* to find the partial derivatives of Equation 1 with respect to *Df*.
$$\mathrm{let},g\left(Df\right)=Acc,h\left(Df\right)=En\times Bs\times \left(1-Df\right)$$

Step 1: Differentiate $g\left(Df\right)$

Since *Acc* is treated as a constant with respect to *Df*:

$$g^{\prime }\left(Df\right)=0$$

Step 2: Differentiate $h\left(En\right)$

$$h\left(Df\right)=En\times Bs\times \left(1-Df\right)$$

Here both *En* and *Bs* are treated constants with respect to *Df*

$$h^{\prime }\left(Df\right)=-En\times Bs$$

$$\frac{\partial f}{\partial Df}=\frac{0\times h\left(Df\right)-g\left(Df\right)\times h^{\prime }\left(Df\right)}{{\left(h\left(Df\right)\right)}^{2}}$$

$$\frac{\partial f}{\partial Df}=\frac{-Acc\left(-En\times Ds\right)}{{\left(En\times Bs\times \left(1-Df\right)\right)}^{2}}$$

Simplify it:

$$\frac{\partial f}{\partial Df}=\frac{Acc}{{\left(1-Df\right)}^{2}}$$

∵derivative is positive

∴Dfincreases whenfdecreases… (Equation 3).

The function appropriately captures the fact that increasing dropout can reduce performance.

Finally, from Equations 1–3 the function is mathematically validated ■.

Our objective is to optimize accuracy while reducing epochs, batch size, and determining the best dropout factor. However, these characteristics are typically interrelated, and there is no simple mathematical equation that explicitly connects them. As a result, we’ll create an objective function that combines all these factors and enables us to reach a compromise between accuracy and resource utilization. Algorithm 1 formalizes the proposed HPO as follows.

HPO is implemented and tested to obtain its result using the Python library (SciPy. Optimize), then feed forward these parameters to Resnet125V2.

**ALGORITHM 1:**
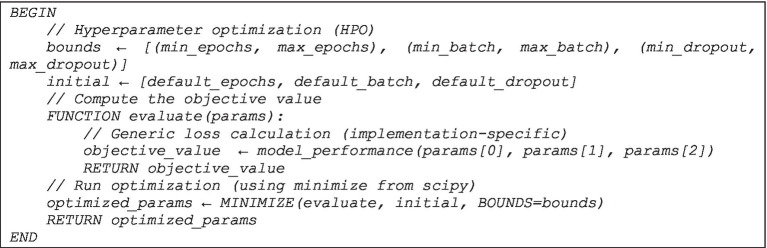
The proposed mathematical model (HPO).

### 3.2 Proposed approach

**Figure 2:**
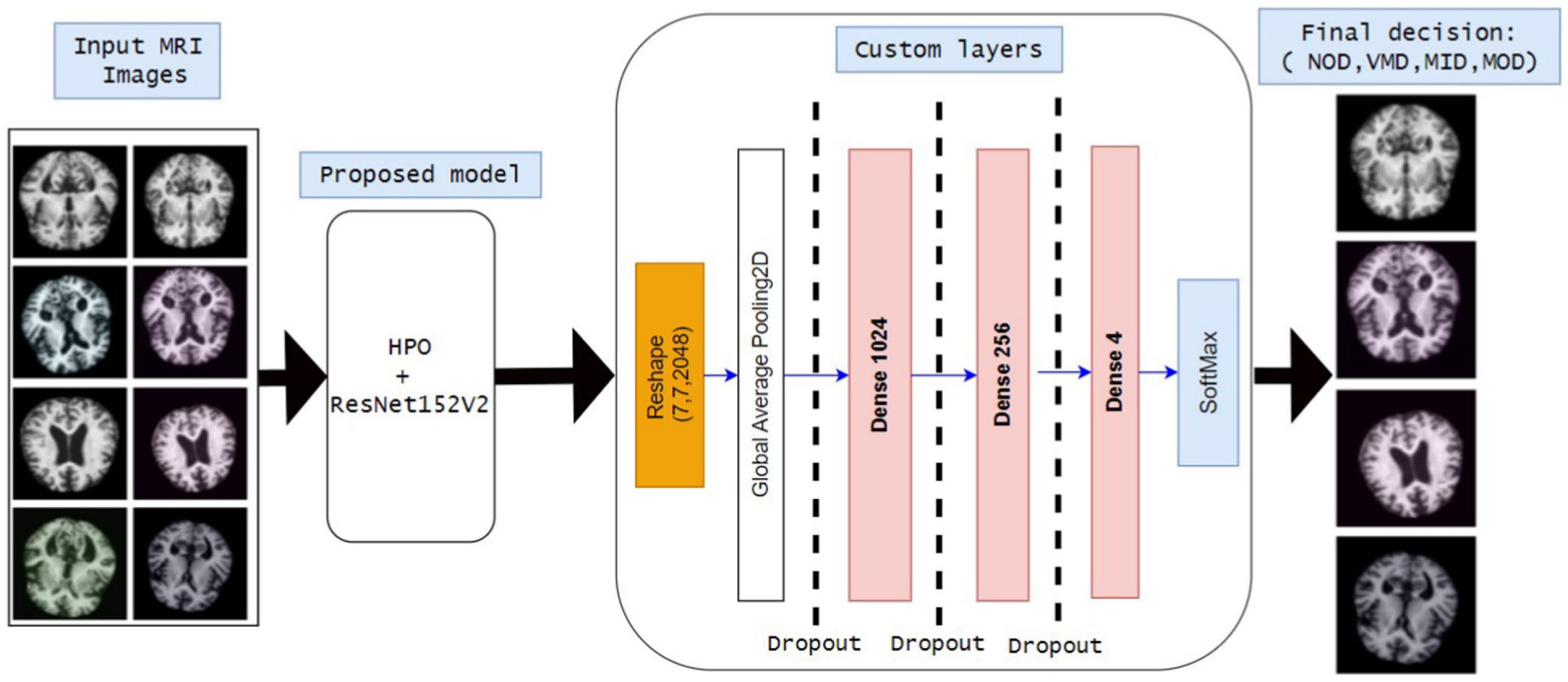
Block diagram of all stages of the proposed model.

Since most deep learning models require a huge amount of computation and resources to achieve the required results, they often use a babysitting approach to adjust their hyperparameters. Moreover, the proposed approach is designed for use in detecting all four phases of AD. The main idea of the proposed approach is to use a pre-trained model (Resnet152v2) after adjusting the hyperparameters (number of epochs, batch size, and dropout factor) using HPO. The workflow of the proposed model is shown in Figure 2:

1. *Reading inputs* as the model takes MRI images as input. The MRI images are classified into four different categories MildDemented (MID), ModerateDemented (MOD), NonDemented (NOD), and VeryMildDemented (VMD).
2. *Proposed Model* (HPO + ResNet152V2)

- HPO is implemented to obtain the best combination of hyperparameters (epochs, batch size, and dropout factor).
- ResNet152V2 is proposed to classify MRI images due to its strength in medical image classifications.

3. *Custom layers*

- Extracted features are reshaped into suitable formats.
- Global average pooling 2D is implemented to reduce dimensionality.
- Three fully connected (Dense) layers are included: Dence 1024, Dence 256, and Dence 4 neurons (represent the final classification classes).

4. *Dropout layers* are included in the model to reduce model complexity, computation overhead, and avoid overfitting.
5. *Final classification layer* (SoftMax activation) to classify entered MRIs into: MID, MOD, NOD, and VMD.

Algorithm 2 formalizes the workflow of the proposed model as follows:

**ALGORITHM 2:**
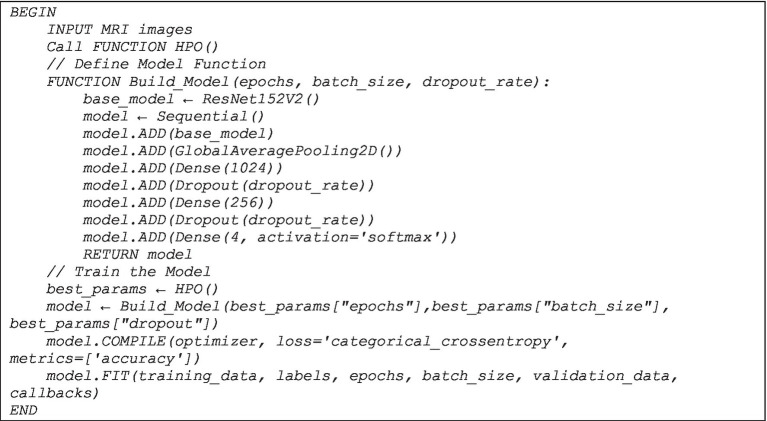
The proposed model (HPO+ResNet152V2).

### 3.3 Dataset preprocessing and preparation

The MRI images are preprocessed to ensure standardization and stability during experiments and to obtain the optimum model performance.

- *Resizing*: all MRI images are resized into (128*128*3) to obtain RGB images using CNN to reach our goal of attaining high performance with low computations and complexity.
- *Scaling*: we change the scale by 1/255 to scale all images from [0,255] to [0,1].

The dataset is considered the most crucial part. Several AD datasets are available online for classification processes, but the known datasets like ADNI and OASIS have some limitations (El-Latif et al., 2023; Liu et al., 2022):

- Publicly not available.
- Huge dataset size.
- Fewer number of samples and classes.
- High computational costs.
- Long processing time and hardware and memory issues.

**Figure 3:**
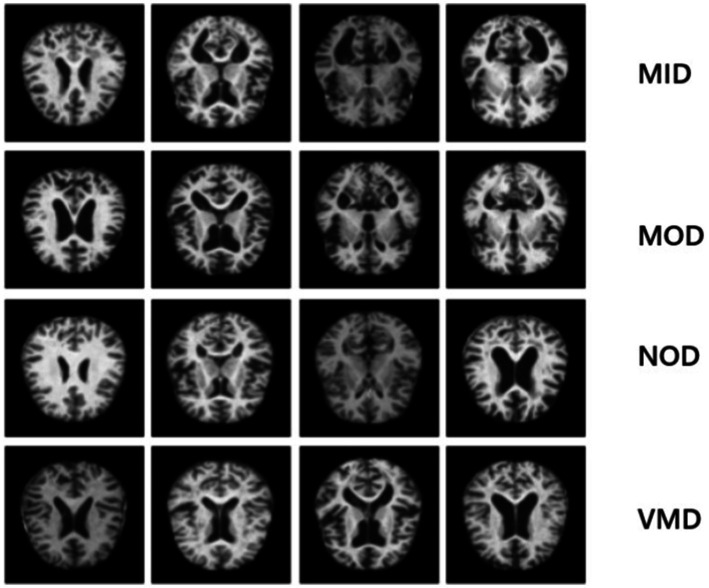
Sample images that illustrate the classes of the disease: the 1st row represents MID class, 2nd row indicates MOD class, 3rd row shows NON class and 4th row represents VMD class.

For these reasons, in this paper, two different datasets obtained from Kaggle were used:

The first dataset (OrDS) was originally curated by Dubey (2020) and subsequently utilized in studies (Ali et al., 2024; El-Latif et al., 2023; Liu et al., 2022). It had about 6,400 MRI; all of them are in jpg format, most of them are 176*208 sizes, that were hand-verified and annotated by experts, obtained from various sources for research purposes, and organized into four directories: MID, MOD, NOD, and VMD. The dataset consisted of 896 MRI for MID, 64 MRI for MOD, 3200 MRI for NOD, and 2,240 MRI for VMD. Sample images are shown in Figure 3 to illustrate the classes. The dataset is publicly available for developing deep-learning models that can effectively classify AD stages, consequently finding the right treatment.

The second dataset (AuDS) was originally curated by Uraninjo (2022) and subsequently utilized in studies (Li et al., 2024; Li, 2024). It comprised MRI scans of Alzheimer’s patients that had been augmented or edited in some way to boost the dataset’s size or diversity and were divided into two directories (train and test). AuDS was divided into four phases: MID, MOD, NOD, and VMD. It had 8,960 MRIs for MID, 6464 MRIs for MOD, 9600 MRIs for NOD, and 8,960 MRIs for VMD. It was created to train deep learning models to identify Alzheimer’s disease. It was an augmented version of OrDS to solve the unbalancing issue of the OrDS. It was labeled with *AuDS* during experiments.

### 3.4 Computational cost

The computational cost of the proposed model depends on:

- *Training complexity:* how the neural network model is trained with the inputs and hyperparameters.
- *Optimization complexity:* how the proposed HPO is trained, scaled, and converged with the hyperparameters.

#### 3.4.1 The computational cost of training

The model complexity depends on the number of training epochs (*En*), batch size (*Bs*), dataset size (N), and model size (MS) as shown in Equation 5.

(5) $$T_{\mathrm{train}}=O\left(\frac{En*N*MS}{Bs}\right)$$

#### 3.4.2 The computational cost of HPO algorithm

The optimization is considered as a black box of solving the objective function and finding the optimum value of hyperparameters, therefore it is supposed to be linear time K denoted by *O(K)*.

$$\mathrm{The total cost}=\left(HPO+\mathrm{Training}\right)$$

The best case as the HPO converges quickly and finds hyperparameters as presented in Equation 6.

(6) $$T_{\mathrm{train}}=O\left(\frac{E*N*MS}{Bs}\right)$$

The worst case: the search space is huge, and the parameters bound are huge as shown in Equation 7.

(7) $$T_{\mathrm{train}}=O\left(K+\frac{E*N*MS}{Bs}\right)$$

## 4 Results and discussion

### 4.1 Experiments setup

The codes and analysis were written and performed using Python with the Jupyter Notebook. Google Colab and Kaggle were used in the training of the ResNet152V2 model and the compilation of Python codes. The experiments were carried out over the two datasets (OrDS and AuDS) under the following considerations:

- The datasets were partitioned into 70% for training and validation and 30% for testing.
- The training partition was divided into 70% for training and 30% for validation.
- Three different optimizers were applied (*Adam, RMSprop, and SGD*) to update the learning rate and processes.
- The SoftMax classifier was used to classify the datasets into four main classes: MID, MOD, NOD, and VMD.
- Two different callbacks were applied to aid in saving the best model, avoiding overfitting, and improving convergence (*ModelCheckpoint, and EarlyStopping*).
- The values of the hyperparameters of model obtained from the HPO are shown in Table 2.

**Table 2:** The values of the hyperparameters that were applied during the experiments.

Hyperparameter	Value
Epochs	50
Batch size	32
Dropout factor	0.2

### 4.2 Performance metrics

These metrics detail how effectively the model performs on an issue, enabling it to judge its strengths and limitations (Hwang et al., 2024; Ibrahim et al., 2021; Powers, 2020; Sethuraman et al., 2023).

*Accuracy:* The ratio of correctly predicted instances to the total number of instances.

- *Formula:*

(8) $$\mathrm{Accuracy}=\frac{\mathrm{Number}\;\mathrm{of}\;\mathrm{Correct}\;\mathrm{Predictions}\;}{\mathrm{Total}\;\mathrm{Number}\;\mathrm{of}\;\mathrm{Rredictions}}$$

*Precision*: Measures the accuracy of positive predictions. High precision means fewer false positives.

- Formula:

(9) $$\mathrm{Precision}=\frac{\mathrm{True}\;\mathrm{Positives}\;}{\mathrm{True}\;\mathrm{Positives}+\mathrm{False}\;\mathrm{Positives}\;}$$

*Recall:* Measures of the ability to identify all positive instances. High recall means fewer false negatives.

- Formula:

(10) $$\mathrm{Recall}=\frac{\mathrm{True}\;\mathrm{Positives}\;}{\mathrm{True Positives}+\mathrm{False Negatives}\;}$$

*F1-score:* Harmonic mean of precision and recall, giving a balance between the two.

- Formula:

(11) $$F1-\mathrm{Score}=2\times \frac{\mathrm{Precision}\times \mathrm{Recall}}{\mathrm{dPrecision}+\mathrm{Recall}}$$

### 4.3 Experiments 1: testing model using OrDS

The model was trained and tested several times over OrDS (6,400 samples), and the dataset was redivided into the training and validation portion (3,135 train, 1,344 validate), and test portion (1,921 sample) using the above-mentioned hyperparameters and configurations by applying three different optimizers SGD, Adam, and RMSprop. The accuracy, precision, recall, and F1-score were calculated using Equations 8–11, respectively. An analysis of the results obtained from different experiments using the SGD, Adam, and RMSprop optimizers is shown in Table 3.

**Table 3:** Results obtained from the proposed model on OrDS.

Optimizer	AD stage	Precision	Recall	F1-score	Train accuracy	Overall accuracy
Adam	MID	0.959	0.877	0.916	94.42%	94.63%
	MOD	0.905	1	0.95		
	NOD	0.954	0.968	0.960		
	VMD	0.932	0.942	0.937		
RMSprop	MID	0.948	0.888	0.917	93.90%	93.80%
	MOD	0.818	0.947	0.878		
	NOD	0.953	0.956	0.955		
	VMD	0.917	0.932	0.924		
SGD	MID	0.947	0.940	0.944	**95.01%**	**95.21%**
	MOD	1	1	1		
	NOD	0.959	0.968	0.963		
	VMD	0.943	0.933	0.938		

The bold values indicate the optimum results obtained during several experiments.

It is shown that the *SGD* optimizer results are the best. The accuracy of classification of the MOD phase reached *100%* accuracy, and the overall accuracy of the model reached *95.21%. SGD* is the optimal choice among the three optimizers, offering the best performance across all AD phases. The SGD achieves perfect precision and recall in the MOD phase, along with high scores in other phases, making it the most robust and effective optimizer. *Adam* is a strong alternative, especially in the NOD and VMD phases, while *RMSprop* lags slightly behind, particularly in the MOD phase. Overall, *SGD* provides the most consistent and reliable results, making it the preferred optimizer for this classification task. Still, the results obtained needed more enhancements as the OrDS was too small to ensure the reliability of the proposed model. The results show that the MOD phase contains a very small number of samples. However, the proposed model was able to achieve satisfactory results in the accurate classification of other phases. Here, to ensure the validity of the proposed model, it was necessary to use a balanced data set containing enough samples in the different disease categories. Accordingly, a balanced dataset was used.

**Figure 4:**
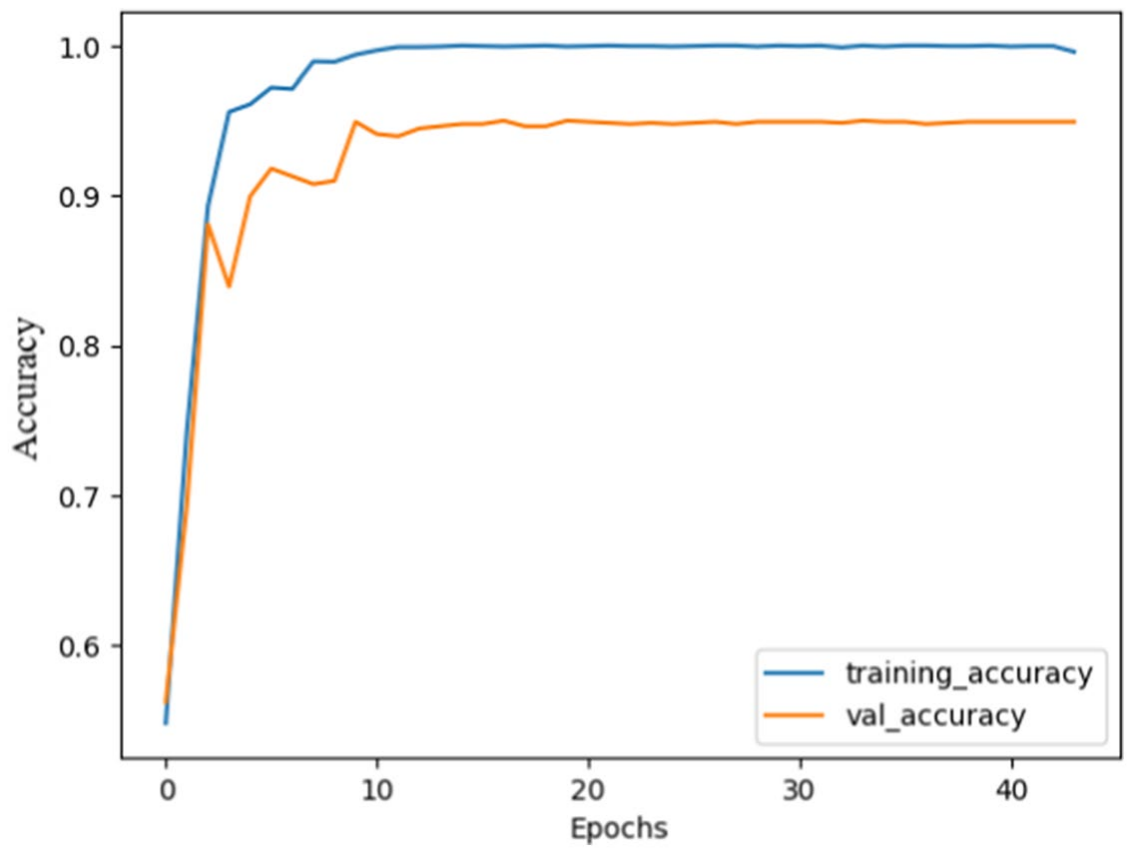
Model accuracy using SGD: the training and validation accuracy increased quickly at the beginning and gradually stabilized after 10 epochs. Both curves almost follow each other, and narrow gap between them, indicating effective model training. Finally, the model curves were almost flattened.

**Figure 5:**
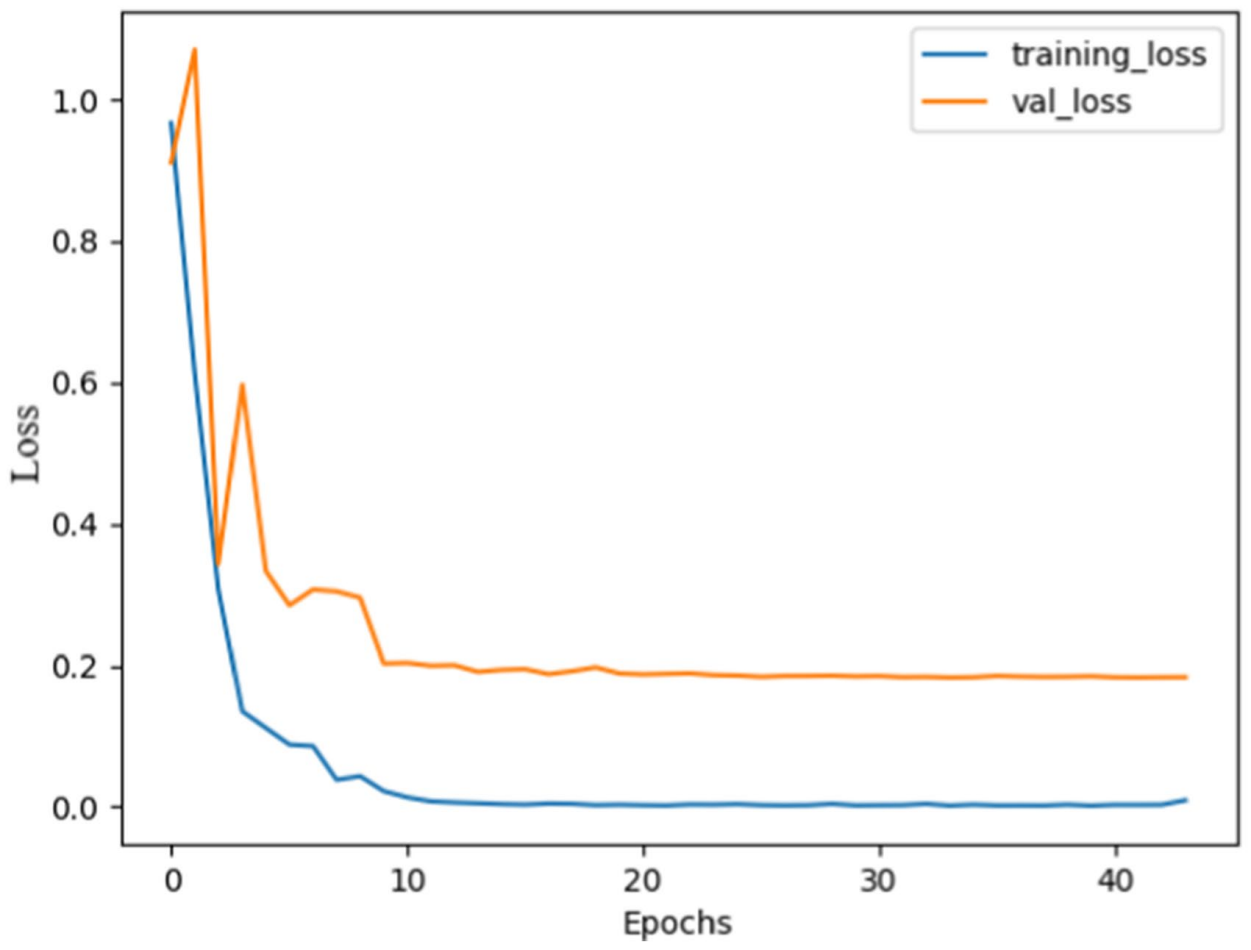
Model loss using SGD: both curves were high, indicating huge mistakes, gradually both curves decreased and stabilized after 10 epochs, with no overshoots observed, the curves almost overlapped after 20 epochs, indicating that the model was not overfitted and effectively recognize patterns.

**Figure 6:**
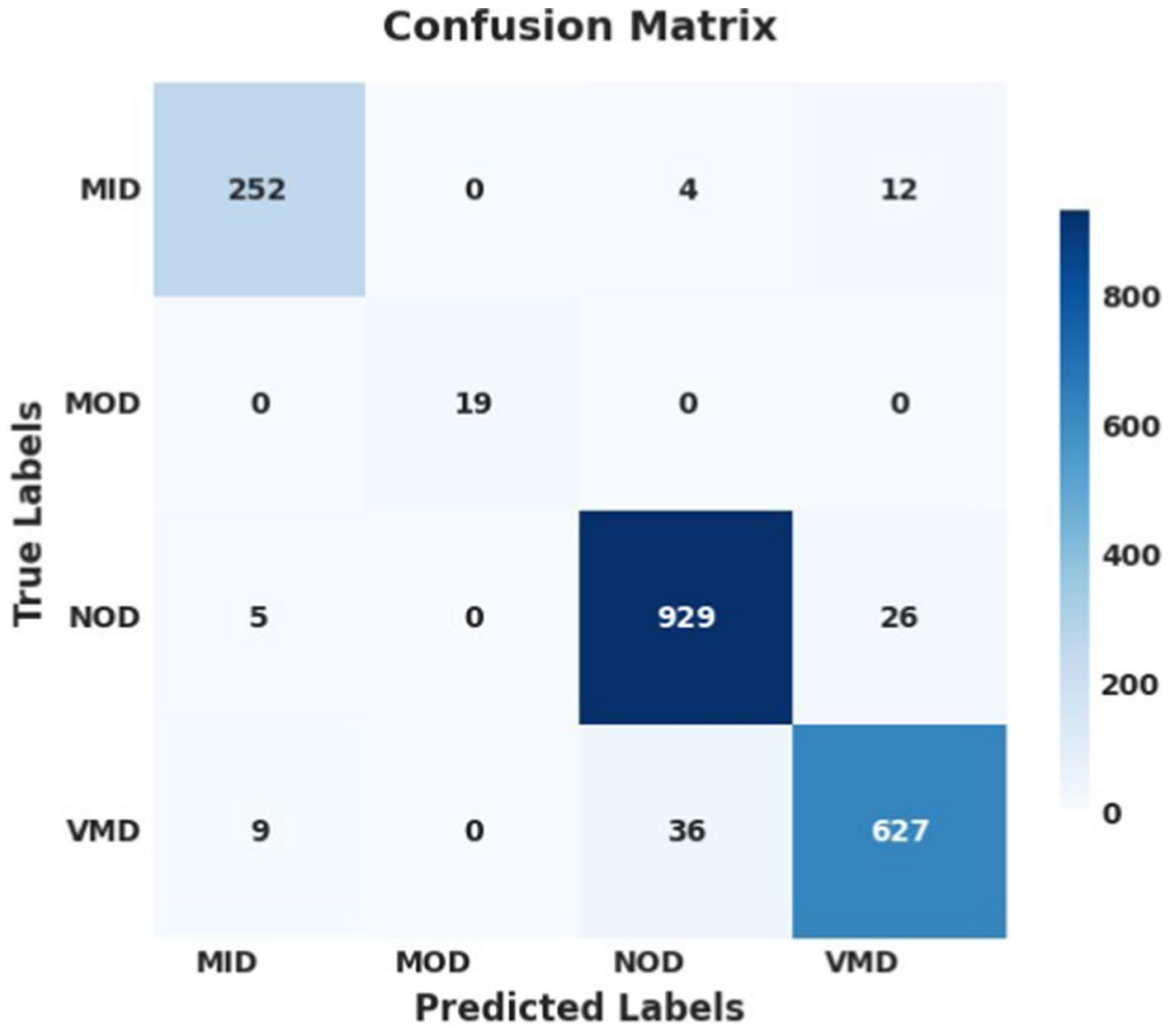
Confusion matrix using SGD optimizer; the model successfully identified and predicted the true labels.

The performance analysis of the proposed model using SGD optimizer during training and validation is shown in Figures 4, 5. The confusion matrix is shown in Figure 6.

The performance analysis of the proposed model during training and validation using Adam is shown in Supplementary Figures 1, 2, and that using RMSprop optimizer is shown in Supplementary Figures 3, 4. The confusion matrices of the proposed model using Adam and RMSprop optimizers are shown in Supplementary Figures 5, 6, respectively.

### 4.4 Experiments 2: testing model using AuDS

The proposed model was tested and trained using AuDS (34,003 samples), the AuDS was partitioned into the training and validation portion (16,661 for training, and 7,141 for validation) and the test portion (10,201 samples). The hypothesis hyperparameters were adjusted and tuned using the HPO model. The results of the experiments are shown in Table 4.

**Table 4:** Results obtained from the proposed model on AuDS.

Optimizer	AD stage	Precision	Recall	F1-score	Train accuracy	Overall accuracy
Adam	MID	0.954	0.948	0.951	92.52%	92.94%
	MOD	0.997	0.999	0.998		
	NOD	0.954	0.858	0.904		
	VMD	0.842	0.937	0.887		
RMSprop	MID	0.976	0.912	0.944	92.93%	92.79%
	MOD	0.999	0.998	0.999		
	NOD	0.933	0.882	0.907		
	VMD	0.837	0.940	0.886		
SGD	MID	0.986	0.993	0.989	**98.56%**	**98.26%**
	MOD	0.999	1	0.999		
	NOD	0.982	0.970	0.976		
	VMD	0.968	0.973	0.971		

The bold values indicate the optimum results obtained during several experiments.

It was noticed from the experiments and the results of the SGD optimizer; the training accuracy was 98.56% and the test accuracy was 98.26%. It classified the AD stages into 4 Phases; it reached an accuracy of 99.9% in the MOD phase, 98.6%in the MID phase, 98.2% in the NOD phase, and 96.8% in the VMD phase.

Adam vs. RMSprop: Both optimizers performed similarly, with Adam slightly edging out in terms of overall accuracy (92.94% vs. 92.79%) and performing better in recall in some phases. However, RMSprop showed slightly better precision in the MID phase. Despite these minor differences, neither Adam nor RMSprop can match the performance of SGD. The results showed that SGD was the most effective optimizer. It delivered the highest precision, recall, and F1-scores across all AD phases and had the highest training and overall accuracy.

This suggested that SGD not only generalizes well to unseen data but also accurately identifies the various stages of Alzheimer’s disease with minimal errors. Therefore, SGD is the optimal choice for this task, consistently outperforming Adam and RMSprop in every phase of AD. It achieves the highest accuracy and balanced performance across all evaluated metrics, making it the most robust and reliable optimizer in this context.

**Figure 7:**
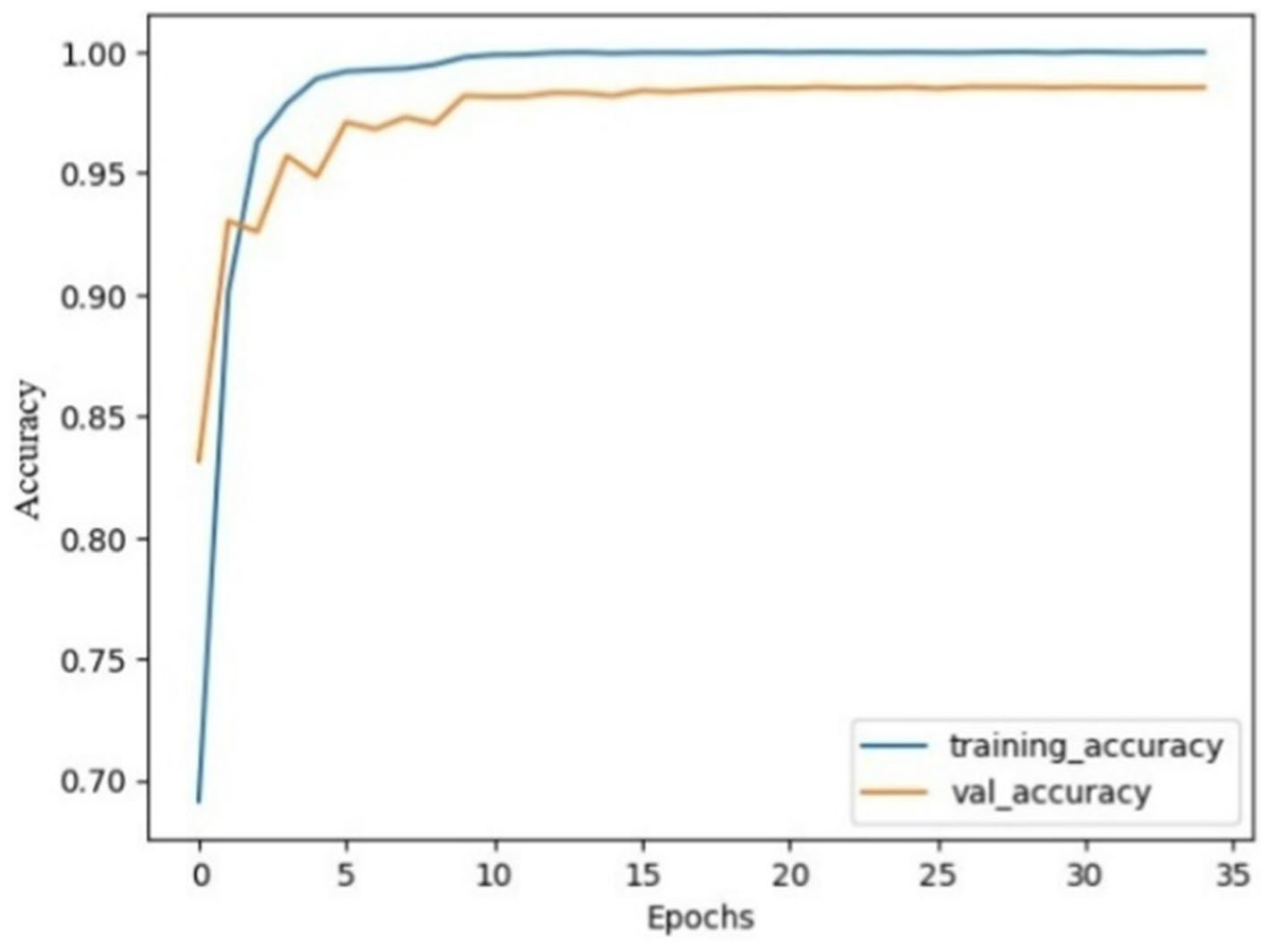
Model accuracy using SGD on AuDS. Both training and validation accuracy rapidly increased from epochs 0 to 5, then smoothed and slowly increased between epochs 5 and 10. Finally, the model stabilized and converged after 10 epochs.

**Figure 8:**
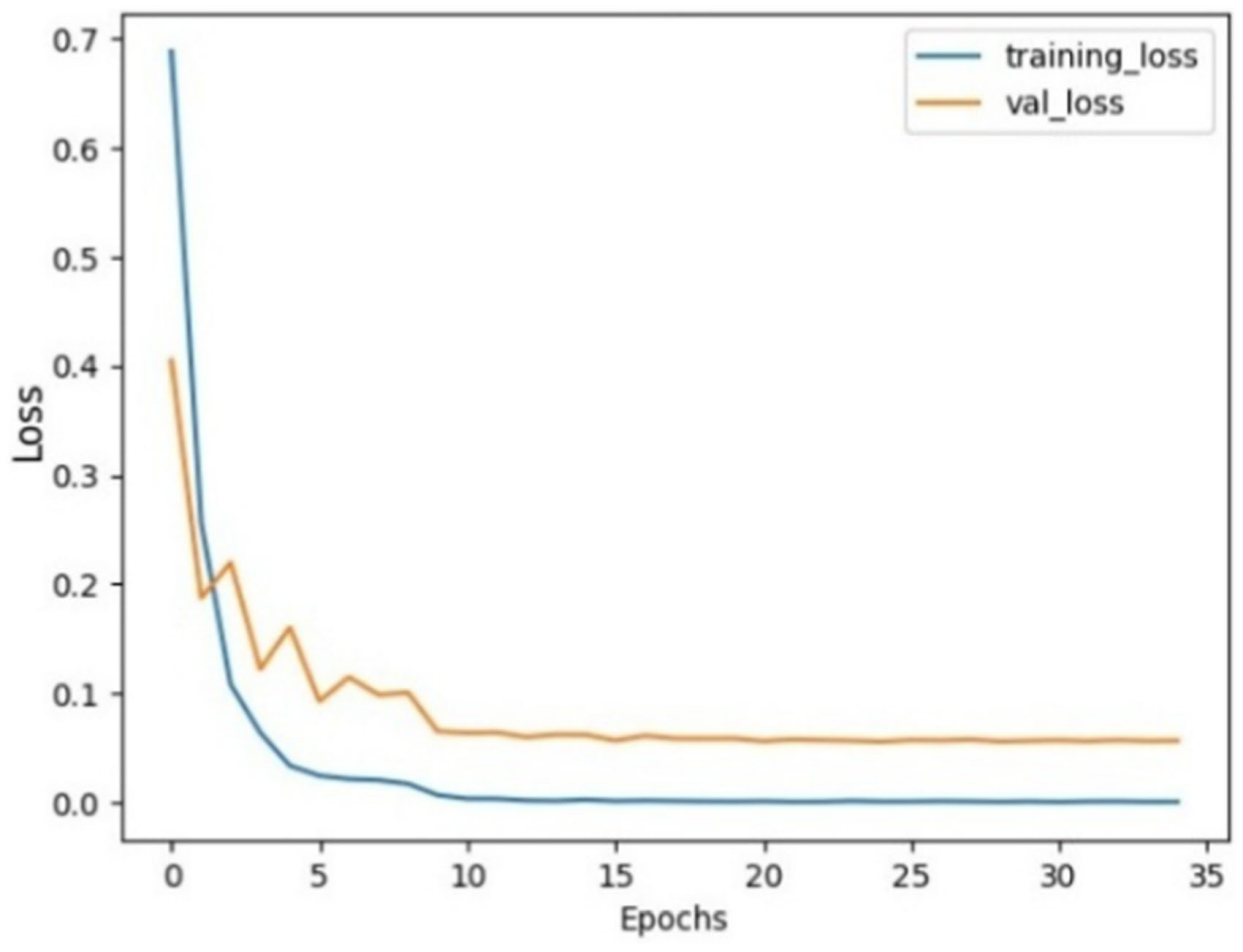
Model loss using SGD on AuDS: Both training loss and validation loss showed a rapid decrease within the initial 5 epochs. Then, validation loss showed a slow decrease between epochs 5 and 15. Finally, both loss curves stabilized and flattened without any sudden overshoot.

**Figure 9:**
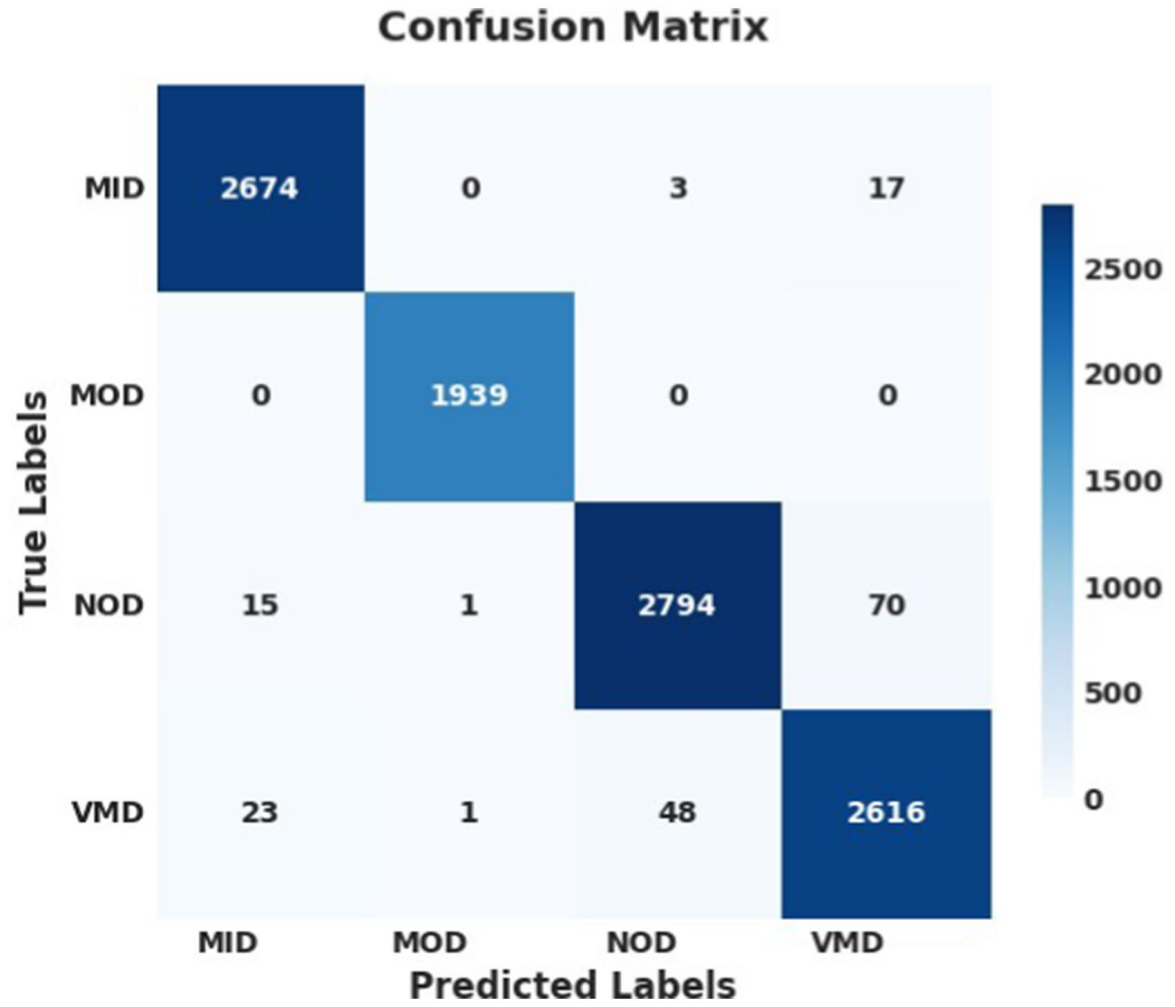
Confusion matrix of the proposed model using SGD optimizer on AuDS.

The performance analysis of the SGD optimizer during training and validation is shown in Figures 7, 8. The confusion matrix on the validation set is presented in Figure 9.

The performance analysis of the proposed model during training and validation using Adam on AuDS is shown in Supplementary Figures 7, 8, and that using RMSprop optimizer on AuDS is shown in Supplementary Figures 9, 10. The confusion matrices of the proposed model using Adam and RMSprop optimizers are shown in Supplementary Figures 11, 12, respectively.

### 4.5 Performance comparison with state-of-the-art models

The suggested approach outperforms existing models in the literature for AD classification as presented in Table 5. This consistent and resilient performance demonstrates the model’s greater capacity to generalize and reliably discriminate across different cognitive levels, which is difficult for many other models. Overall, the suggested approach represents a significant leap in AD diagnosis, delivering more accurate and effective categorization than the current literature.

**Table 5:** Performance analysis of the proposed model versus state-of-the-art-models.

Authors	Model	Database	Classification method	Accuracy
El-Latif et al. (2023)	Lightweight CNN	OrDS	Binary and multi classifier.	Binary classification: 99.22%; multi classification: 95.93%.
Sethuraman et al. (2023)	CNN, ALEXNET, inception blocks	ADNI	Binary classifier: AD and Normal control (NC)	96.61%
Hu et al. (2023)	VGG-16 + transformer	ADNI	Multi classifier	77.20%
Mora-Rubio et al. (2023)	CNN, pre-trained deep learning models	ADNI+ OASIS	Multi classifier: NC, and Mild Cognitive Impairment (MCI),	89% AD vs. NC 80% Late MCI vs. NC 66% MCI vs. NC 67% Early MCI vs. NC
Hazarika et al. (2023)	Hyper model (LetNet+ AlexNet)	ADNI	Multi classifier: CN, MCI, and AD	93.58%
Shojaei et al. (2023)	3D CNN	ADNI	Binary classifier: AD vs. NC	96%
Jraba et al., 2024	VGG-16 Inception-v3 ResNet152	Images collected from different resources	Multi classifier: NOD, VMD, MID, and MOD	98% for ResNet 152, 96% for Inception-v3, and 80% for VGG 16
Nagarathna et al. (2023)	Deep Learning (Multi-layer Feed-forward Neural Network)	Collected from ADNI+ Kaggle datasets	Multi classifier: AD, MCI, and NC	93.38%
Shaziya and Zaheer (2021)	Machine Learning (Logistic Regression, SVM, Extreme Gradient Boosting, MLP)	PET images from 199 participants.	Multi classifier: cognitive unimpairment (CU), MCI, and AD	AUC > 0.96 for CU vs. AD; AUC 0.88 for MCI vs. AD; AUC 0.75 for MCI vs. CU
Tuvshinjargal and Hwang (2022)	Pre-trained model	Kaggle	Predict AD using MRI dataset	77.46%
Ghazal and Issa (2022)	Utilized transfer learning	OrDS	Multi classifier: MID, NOD, MOD, and VMD	91.70%
Li et al. (2024)	a novel adaptive disease detection model named EAFP-Med ST	AuDS	Propose EAFP-Med, an efficient adaptive feature processing module based on prompts for medical image detection	97.60%
Our work	Proposed Model	AuDS	Multi classifier: MID, NOD, MOD and VMD	**98.26%**

The bold values indicate the optimum results obtained during several experiments.

In terms of results, the table showcases various approaches to AD classification, with different models, data sources, and classification strategies. Most models focus on binary classification between AD and NC, with accuracy generally above 90%. Multi-class classifiers show more variation in accuracy, depending on the classes being distinguished. The proposed model in our work stands out with a 98.26% accuracy in multi-class classification of different AD stages, surpassing most other models, particularly in multi-class scenarios, especially when using stochastic SGD. Unlike previous models that frequently use optimizers such as Adam and RMSprop, the suggested model yields greater precision, recall, and F1 scores throughout all AD phases, with perfect accuracy in the MOD phase.

### 4.6 Ablation analysis

In this part, several trials and analyses of the proposed model were performed to ensure its strength and dependability.

- Due to budget limitations, the trials were trained using GPU T4 on Kaggle and Google Colab platforms.
- Data splitting was 70% for training, 20% for validation, and 10% for testing.
- AuDS was the proposed dataset.

The ablation analyses were performed on three strategies: unplanned hyperparameters, planned hyperparameters, and combined ablation.

#### 4.6.1 Unplanned hyperparameters ablation

In this strategy, the hyperparameters: epoch numbers, batch size, and dropout factor were changed in a randomized form. Several experiments were performed on the ResNet152V2 model, and the results are shown in Table 6. Table 6 presents the results obtained from various trials to evaluate the performance of the proposed model using different values of hyperparameters. The trials were performed with several values of hyperparameters (epoch numbers, batch size, and dropout factor). The performance metrics were training accuracy and testing accuracy.

**Table 6:** Results from running the ResNet152v2 with different hyperparameters values.

Trial No.	Batch size	Dropout	Epochs	Train accuracy (%)	Test accuracy (%)
1	128	0.6	150	18.78	19.01
2	64	0.58	100	26.07	26.35
3	64	0.55	250	26.56	26.35
4	32	0.5	200	95.66	95.67
**5**	**32**	**0.2**	**50**	**98.56**	**98.26**
6	16	0.35	120	96.43	96.58
7	8	0.7	350	99.95	94.11
8	8	0.1	180	100	97.84
9	4	0.40	300	98.12	94.21
10	4	0.25	220	99.98	95.04
11	2	0.5	160	67.78	66.42

The bold values indicate the optimum results obtained during several experiments.

The key observations were:

*Impact of batch size*: small batch sizes showed higher training accuracy, like trial 8, which reached a training accuracy of 100%, but the test accuracy was 97.84%, indicating it might be overfitted. On the other hand, large batch sizes had bad test and train accuracy like trail 1.
*Effect of dropout:* a high dropout value (0.6) had low accuracy. However, a low dropout value (0.1) had better accuracy. Finally, moderate dropout values (ranging from 0.35 to 0.5) balanced between model accuracy and avoiding overfitting.
*Epochs:* a high number of epochs did not ensure better results. For example, in trial 7, the epochs were 350 and the model overfitted.

In summary, the results indicate that finding the best combination of hyperparameters is crucial for the model’s performance. Finally, it is noticed from the results that trial 5 with 32 batch sizes with 0.2 dropout and 50 epochs reached the optimum model accuracies and performance. These values are equal to the values obtained from the proposed HPO model.

#### 4.6.2 Planned hyperparameters ablation

In this strategy, the proposed model was trained and tested several times using values of epoch numbers, batch size, and dropout obtained from the HPO model. The experiments were performed by changing the values of one of the hyperparameters and fixing the others, studying its effect on the proposed model. The results were summarized in Tables 7–9. The performance metrics were train and test accuracy, precision, recall, and F1-score.

**Table 7:** Results from ResNet152v2 model where dropout = 0.2 and batch size = 32.

Epochs	Train accuracy	Test accuracy	Precision	Recall	F1-score
10	0.82	0.80	0.83	0.81	0.80
20	0.91	0.93	0.93	0.93	0.93
30	0.96	0.97	0.97	0.97	0.97
40	0.98	0.98	0.98	0.98	0.98
**50**	**0.99**	**0.98**	**0.98**	**0.98**	**0.98**

The bold values indicate the optimum results obtained during several experiments.

**Table 8:** Results from ResNet152v2 model where epochs = 50 and batch size = 32.

Dropout	Train accuracy	Test accuracy	Precision	Recall	F1-score
0.1	0.98	0.98	0.99	0.98	0.98
**0.2**	**0.99**	**0.98**	**0.98**	**0.98**	**0.98**
0.3	0.28	0.28	0.07	0.25	0.11
0.5	0.90	0.92	0.93	0.92	0.92
0.7	0.97	0.97	0.97	0.97	0.97
0.8	0.96	0.98	0.98	0.98	0.98
0.9	0.95	0.96	0.97	0.96	0.96

The bold values indicate the optimum results obtained during several experiments.

**Table 9:** Results from ResNet152v2 model where epochs = 50 and dropout = 0.2.

Batch size	Train accuracy	Test accuracy	Precision	Recall	F1-score
2	0.26	0.26	0.07	0.25	0.10
4	0.82	0.84	0.85	0.85	0.85
8	0.77	0.78	0.80	0.80	0.80
16	0.93	0.94	0.95	0.95	0.95
**32**	**0.99**	**0.98**	**0.98**	**0.98**	**0.98**

The bold values indicate the optimum results obtained during several experiments.

Table 7 shows how the performance of the model was improved as it was trained for more epochs. It was noticed from the table that the model performance improved while the training epochs increased, as noticed from the evaluation metrics. Finally, from epochs 40 and 50, the model’s performance reached optimality, and no overfitting was noticed, showing the model generalizes well to new data. Training for 50 epochs was enough and ideal since there were no more improvements after this point. The model was effective and efficient.

In Table 8 the epoch number and batch size were fixed to values 50 and 32, respectively, while varying the dropout value. The result shows the performance improvement of the model concerning the changing dropout values. It is noticed that with small dropout values like 0.1 and 0.2, the performance metrics were high, and no overfitting was noticed. At dropout 0.3, the performance collapsed, and the model struggled to make meaningful predictions. While high dropout values performed well, the performance metrics decreased and slowed down the learning. Finally, the dropout of 0.2 was ideal for this task, balancing learning and generalization.

In Table 9 the epoch number and dropout were fixed to values 50 and 0.2, respectively, while varying the batch size value. The model performance improvement concerning changing batch size values. As noticed from experiments, when the batch size was too small, like 2, the performance metrics were too small, and the model could not provide meaningful results. In addition, when the batch size increased, the performance improved gradually. Finally, the optimum batch size value was 32 when the performance metrics were near-perfect results.

In conclusion, Tables 7–9 present the performance metrics of the ResNet152V2 model under different configurations of epochs, batch size, and dropout. The results show how the model performs for changing the values of these variables. Moreover, finding the balanced values of epochs, batch size, and dropout is critical for the model’s performance. In addition, the best settings were 50 epochs, 32 batch sizes, and 0.2 dropouts, which gave the highest accuracies and performance and ensured the stability of our proposed HPO model.

#### 4.6.3 Combined ablation

In this part of the analysis, the proposed HPO model was tested and validated with various transfer models.

The following considerations were proposed:

- The hyperparameter values obtained from the HPO model were 50 epochs, 32 for the batch size, and 0.2 for the dropout.
- The key performance metrics were training and testing accuracy, precision, recall, and F1 score. The results were summarized and illustrated in Table 10.

**Table 10:** Results obtained from several models using mathematical model (HPO) hyperparameters value; epochs = 50, batch size = 32, and dropout = 0.2.

Model	Train accuracy	Test accuracy	Precision	Recall	F1-score
MobileNet	0.66	0.66	0.7	0.67	0.68
MobileNetV2	0.61	0.56	0.61	0.56	0.56
EfficientNetB0	0.28	0.28	0.12	0.25	0.12
EfficientNetB1	0.28	0.28	0.07	0.25	0.11
EfficientNetB2	0.28	0.28	0.07	0.25	0.11
EfficientNetB3	0.29	0.27	0.15	0.26	0.15
EfficientNetB4	0.29	0.31	0.15	0.28	0.19
EfficientNetB5	0.26	0.26	0.07	0.25	0.10
EfficientNetB6	0.28	0.28	0.15	0.25	0.11
EfficientNetB7	0.28	0.28	0.07	0.25	0.11
DenseNet121	0.58	0.58	0.58	0.59	0.56
DenseNet169	0.61	0.58	0.62	0.59	0.58
DenseNet201	0.62	0.58	0.61	0.59	0.57
ResNet50	0.61	0.64	0.66	0.67	0.66
**ResNet50V2**	**0.99**	**0.99**	**0.99**	**0.99**	**0.99**
ResNet101	0.66	0.67	0.68	0.69	0.68
**ResNet101V2**	**0.98**	**0.99**	**0.99**	**0.99**	**0.99**
ResNet152	0.78	0.80	0.82	0.82	0.82
**ResNet152V2**	**0.99**	**0.98**	**0.98**	**0.98**	**0.98**
VGG19	0.53	0.56	0.55	0.58	0.56
VGG16	0.56	0.59	0.59	0.61	0.59

The bold values indicate the optimum results obtained during several experiments.

Table 10 shows how the different transfer learning models perform when classifying AD. As noticed from experiments, performance metrics vary as follows:

- The best performance was achieved in ResNetV2 models, especially ResNet50V2, ResNet101V2, and ResNet152V2. These models reached high scores in accuracy, precision, recall, and F1-score, leading to them being the most suitable models for AD classification.
- DenseNet models were accepted but not competitive with ResNetV2 models.
- VGG models were not accepted for the AD classification due to their old architecture.
- EfficientNet models were not suitable at all for this task as they might require fine-tuning.
- MobileNet models might be accepted in case of resource limitations.

In addition, it can be concluded that the proposed mathematical model (HPO model) outperforms traditional methods for fine-tuning hyperparameters for the ResNetV2 models (50 V2, 101 V2, 152 V2). As they are the best choices for high accuracy and reliable predictions.

Finally, it can be concluded that the proposed mathematical model (HPO model), which is proposed to find the hyperparameters of the transfer learning models, is robust and suitable for AD classification when applied with ResNetV2 models. Moreover, the proposed approach finds the optimum results under various experiments with AD.

## 5 Conclusion

In this study, a new optimization method (HPO) for selecting the best hyperparameter values to achieve better accuracy is applied over ResNet152V2. The HPO proved its strength against different optimizers Adam, SGD, and RMSprop. It is tested on AD to classify its different stages. The method needs to be applied and tested on other benchmarks. It is recommended that this model be used in medical device terminals to make real-time classification.

The major drawbacks that face such models are the training process, a huge number of computations that exceed the ability of ordinary machines, and dataset availability. For these reasons, research uses Kaggle and Google Colab platforms to perform computations to achieve better training and results. For dataset availability, it is recommended to collect more real-world MRIs.

The proposed approach can be used to enhance hyperparameter values for better accuracy. This method can be extended or used with other models. AD was classified accurately into four different stages exceeding ordinary methods that distinguish between AD and other brain diseases. In future work, we want to generalize and validate the HPO model against different diseases. Moreover, applying naturally inspired algorithms like genetic algorithms, ant colony, simulating annealing, coco search, and differential evolution to solve the objective function of the HPO model.

## Data Availability

Publicly available datasets were analyzed in this study. This data can be found at: https://www.kaggle.com/datasets/uraninjo/augmented-alzheimer-mri-dataset-v2.
