# Supplementary material for: A novel deep learning technique for multi classify Alzheimer disease: hyperparameter optimization technique
Source: Front Artif Intell. 2025 Apr 24;8:1558725. doi: 10.3389/frai.2025.1558725 (PMC12058654; doi:10.3389/frai.2025.1558725)
Supplement: Supplementary file 1 [file Data_Sheet_1.PDF]

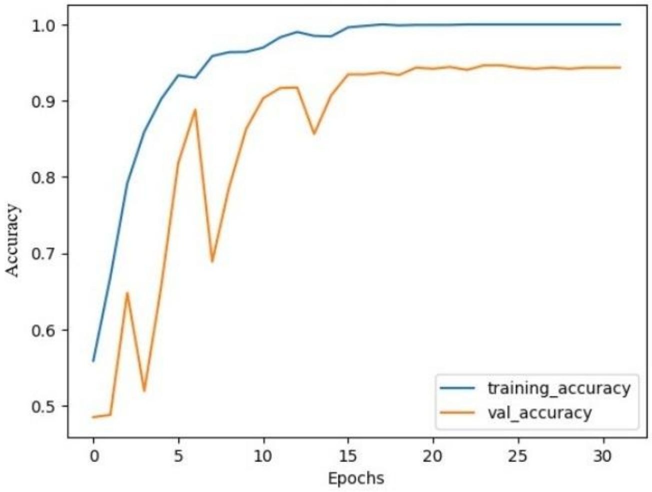

SUPPLEMENTARY FIGURE 1 Model accuracy using Adam optimizer on OrDS: The training accuracy gradually increased and stabilized after 15 epochs. However, the validation accuracy has three phases: in the initial phase (epochs 0-5), the curve increased gradually; in the second phase, (epochs 5-15), the curve steadily increased and decreased; and in the third phase (after 115 epochs), the curve stabilized and converged without any drastic drop.

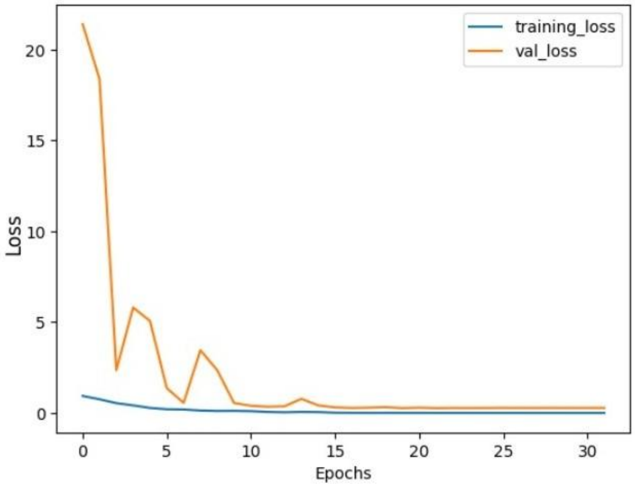

SUPPLEMENTARY FIGURE 2 Model loss using Adam optimizer on OrDS: The training loss and validation loss both dropped quickly within 5 epochs. The training loss entered steady improvement from epochs 5 to 15, and both curves flattened after 15 epochs. On the other hand, validation loss battled improvement from epochs 5 to 15.

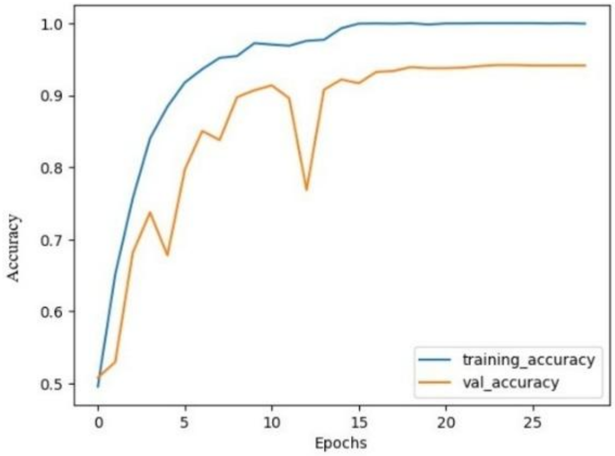

SUPPLEMENTARY FIGURE 3 Model accuracy using RMSprop optimizer on OrDS: The training accuracy gradually increased and stabilized after 15 epochs. However, validation accuracy rapidly increased from epochs 0 to 5, then struggled between increasing and decreasing from epochs 5 to 15, and reached stability and converged after 15 epochs.

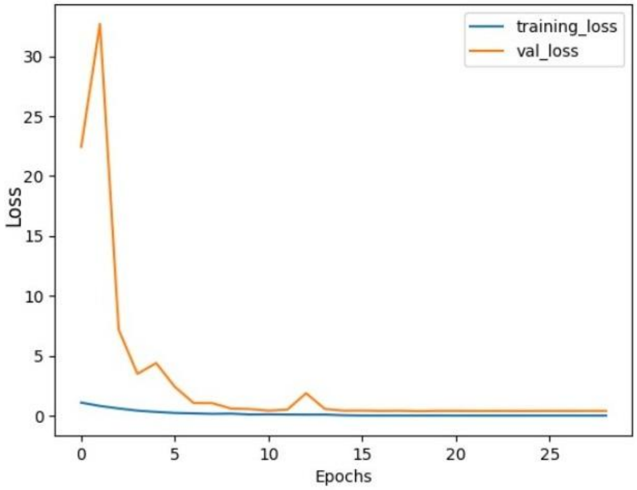

SUPPLEMENTARY FIGURE 4 Model loss using RMSprop optimizer on OrDS: The training loss stabilized early by epoch 5 without any sudden overshot. Conversely, validation loss overfitted and learned training data from epochs 10 to 15, improved gradually between epochs 5 and 10, and then rapidly decreased between epochs 0 and 5. This occurred because the optimizer used lacked momentum, unlike Adam. As a result, they are stuck in local optima.

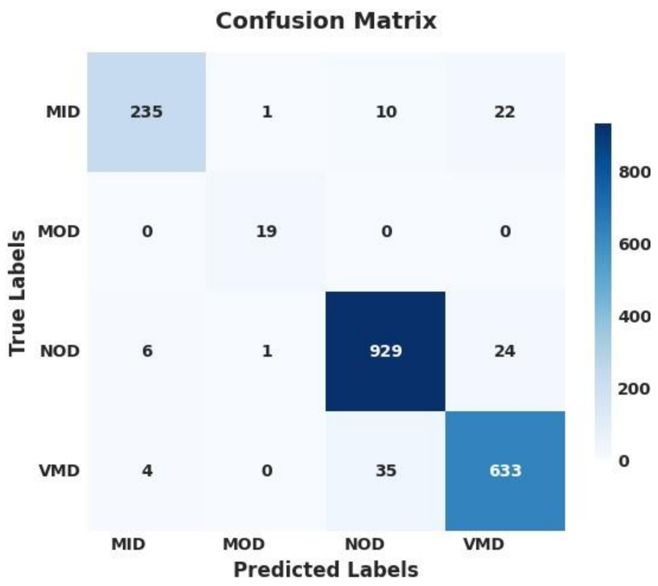

SUPPLEMENTARY FIGURE 5 Confusion matrix using Adam optimizer, the model successfully identified and predicted the true labels

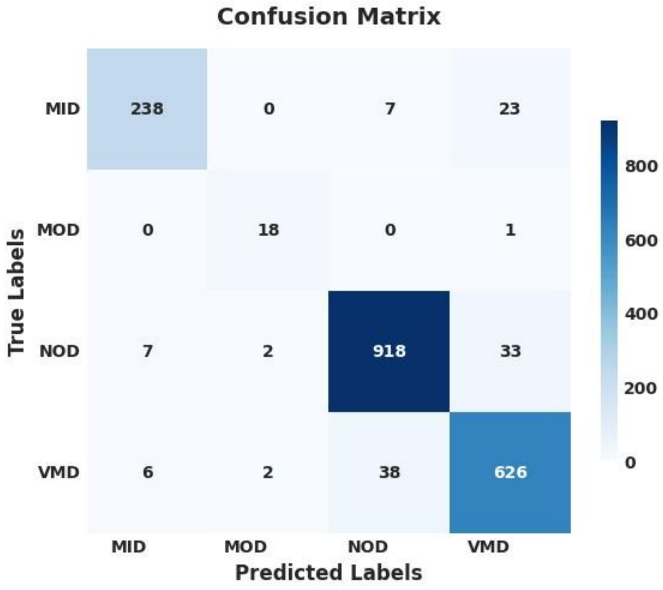

SUPPLEMENTARY FIGURE 6 Confusion matrix using RMSprop optimizer: the model successfully identified and predicted the true labels with some mislabeled in some classes.

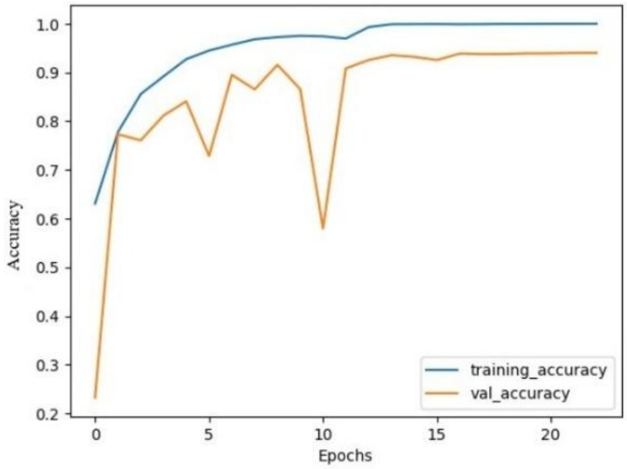

SUPPLEMENTARY FIGURE 7 Model Accuracy using Adam on AuDS: Both training and validation accuracies increased rapidly within the initial 5 epochs. Then, the validation accuracy struggled to identify the new data from epoch 5 to 12 with a sudden drop around epoch 10 indicating the model wasn't able to maintain consistency on the validation set. Finally, it was noticed the model might be overfitted after 15 epochs as the gap between curves increased.

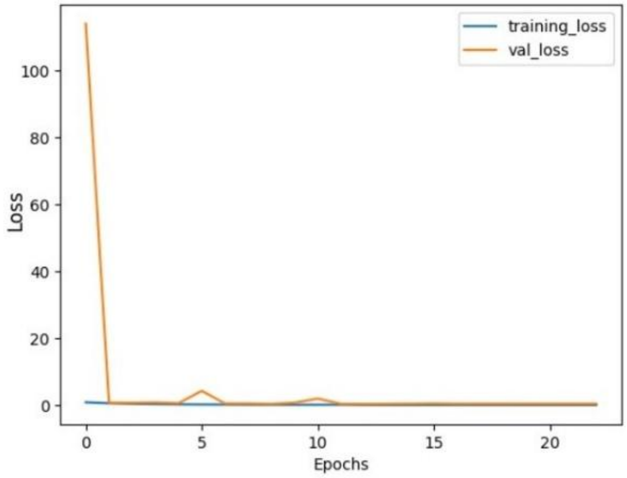

Supplementary Figure 8 Model loss using Adam optimizer on AuDS: The validation started very high and dropped rapidly within the initial few epochs. Both training and validation losses became very small and overlapped after 10 epochs, considering slight struggles with the validation loss around epochs 5 and 10. Finally, the loss curves indicated the model might be overfitted and had memorized training data.

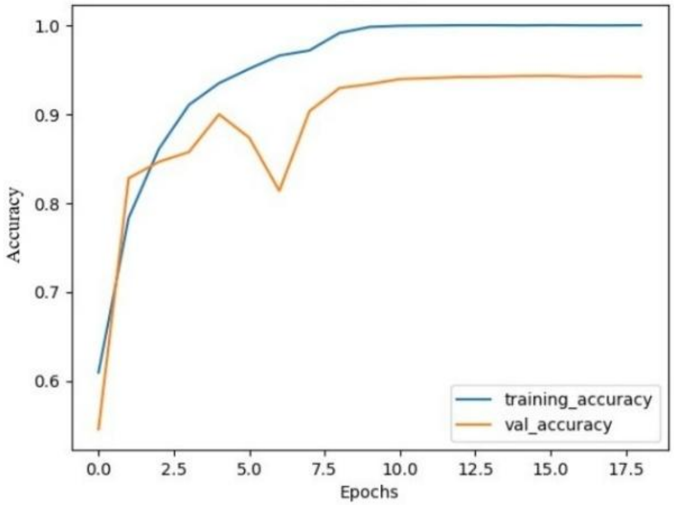

Supplementary Figure 9 Model accuracy using RMSprop on AuDS: Both the training and validation accuracies increased quickly within the initial epochs; the training accuracy stabilized and converged after 10 epochs. Unlike the validation accuracy, it struggled between epochs 5 with a sudden drop occurring around epoch 7. Finally, both accuracies stabilized with an observed gap between them indicating the probability of overfitting.

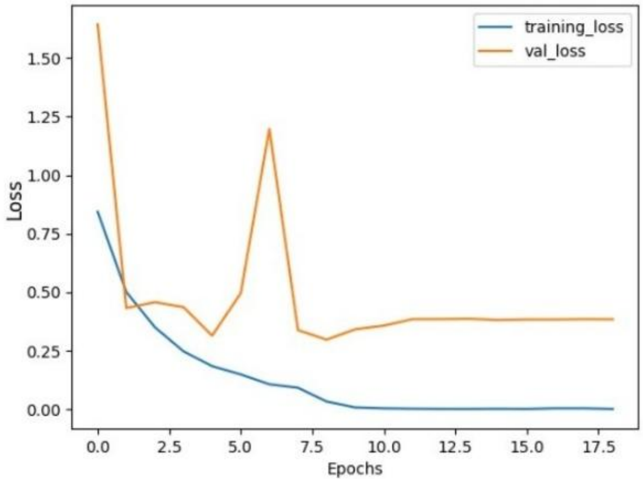

Supplementary Figure 10 Model loss using RMSprop on AuDS: The training loss improved well, indicating the model learned well from data, and it stabilized and converged near zero after 10 epochs. On the other hand, validation loss was unstable: firstly, it started high and decreased within the initial epochs. Secondly, a sudden overshoot happened around epochs 5 and 7, indicating it struggled with some data. Finally, the validation loss didn't improve much with an observed gap between validation and training loss, which is a classical sign of overfitting.

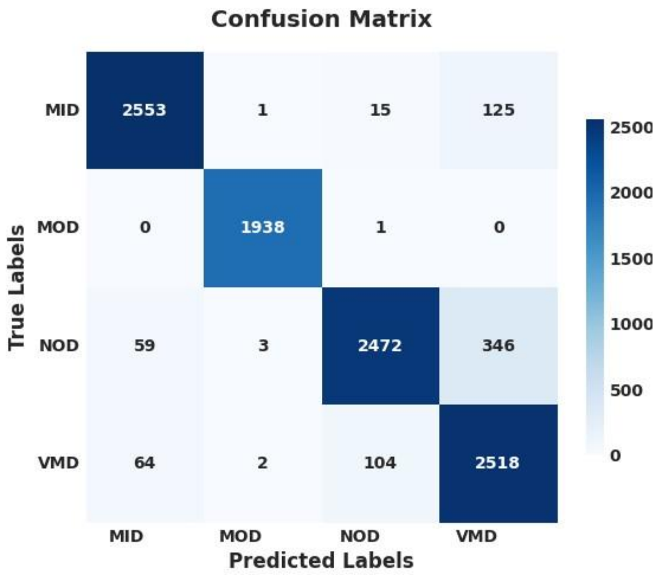

Supplementary Figure 11 Confusion matrix on AuDS using Adam optimizer: The MOD class was nearly accurately identified by the model. The MID and NOD classes performed well, but they lacked sufficient accuracy. The VMD class was the worst.

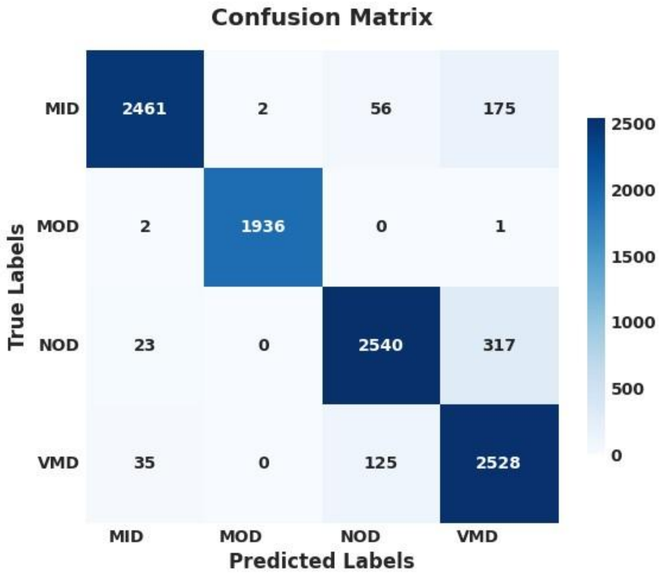

Supplementary Figure 12 Confusion matrix on AuDS using RMSprop optimizer: The model successfully identified the true labels. However, some misclassifications occurred, especially in the NOD and VMD, as these phases have some similar features.
